# Supplementary material for: Polymorphisms in Pfkelch13 domains before and after the introduction of artemisinin-based combination therapy in Southwest Nigeria
Source: PLoS One. 2025 Mar 31;20(3):e0316479. doi: 10.1371/journal.pone.0316479 (PMC11957316; doi:10.1371/journal.pone.0316479)

Samples: 12799
Bases: 921
Average spacing: 14

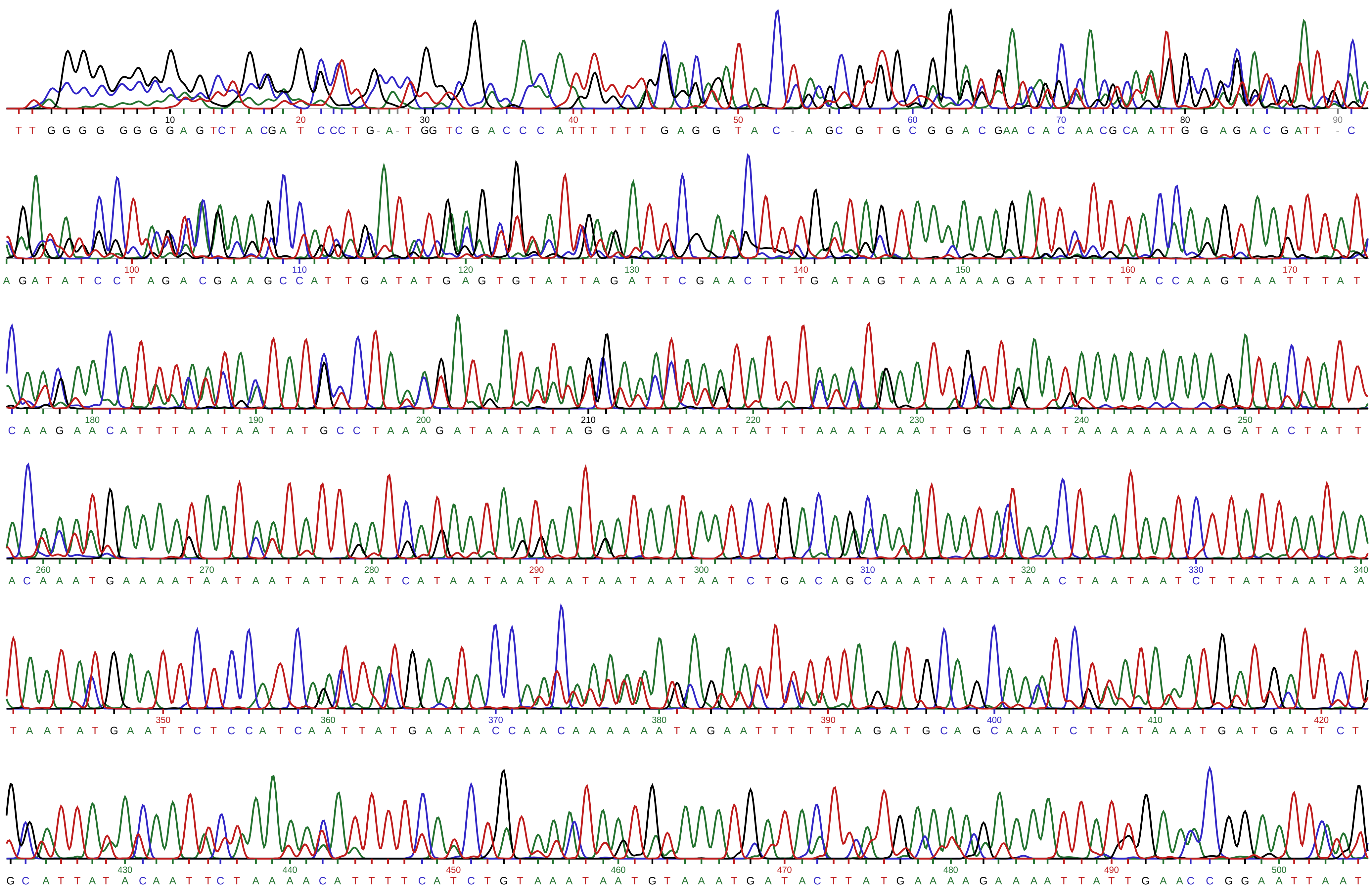

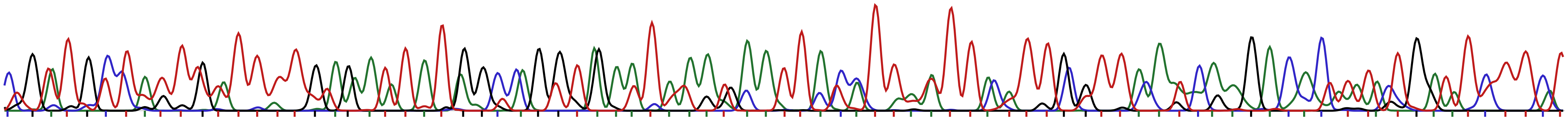

510 520 530 540 550 560 570 580

C G A T G C T A T T G T T T T T G A G A T T A T G G C A G G T A A A T A A A T A A T T A C A T T A A T T A T T T G G T T A A T C A A G A C A C T T A T G A A T C T T C T

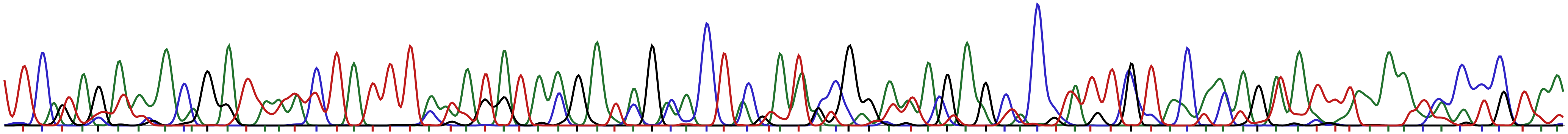

590 600 610 620 630 640 650 660 670

T C T A G A A A C A G A T A A T C T A T T T A T A T A T A A G A T A G C A C T C T A T A C G G A T A G A G C A C T A T T G T A C A A A G A A T T T A A A C A C C C T A A

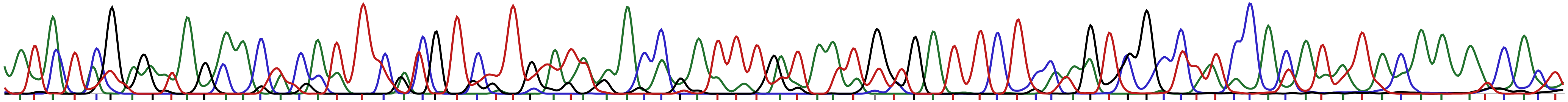

680 690 700 710 720 730 740 750

A T A T C G A G T A G A A C A C A T T C A C G T C T T G A T A A A C C G A T T T A T A A T - T G A T T C T C C A G T G G C C T T C C A C A T A T A C A A A T C A C T

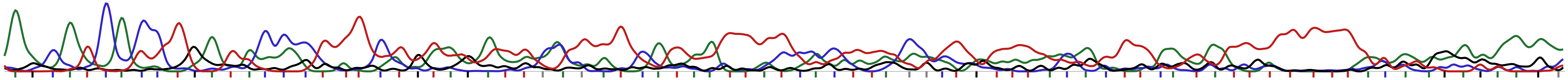

760 770 780 790 800 810 820 830 840

A G C A T C A C C T G A T A C C C T T T C T G T A G A T T - A - A T C A T A A T T A T C A C T T A - - C T G T T T A C A C T T C A T A T T T T T T T A C A A G A G A A G -

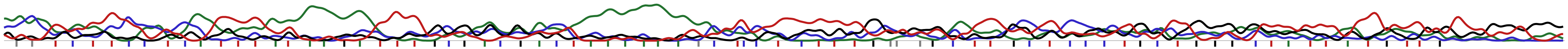

850 860 870 880 890 900 910 920

- - T C T T C C T C A T T T A T A A G A T T T G C G A C G A C T A A A A A A - C T C C T C T T T G - - - G C T T G C T C C C T G C T G G G C T A T T A T G T T G

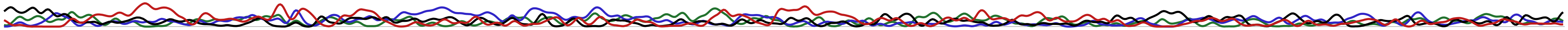

Samples: 12799
Bases: 921
Average spacing: 14

8/17/2022

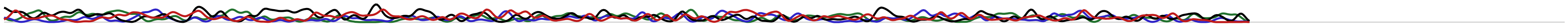

Supplement: Supporting information 2 — (ZIP) [file pone.0316479.s002.zip › 198KN2F_PREMIX_Plate_KELCH2_C08.pdf]
